# Skin Biophysical Parameters and Patch Test Results in People Predisposing to Xiaotong Tiegao Induced Irritant Contact Dermatitis

**DOI:** 10.1155/2019/8612561

**Published:** 2019-02-24

**Authors:** Hai-yan Cheng, Lin-feng Li

**Affiliations:** Department of Dermatology, Beijing Friendship Hospital, Capital Medical University, 95 Yong An Road, Xi Cheng District, Beijing 100050, China

## Abstract

**Background:**

Xiaotong Tiegao (XTT) is an ancient topical Tibetan medicine plaster which is widely used in China. Irritant contact dermatitis (ICD) caused by XTT is very common. It is still unclear why some people are more prone to develop ICD. The aim of this study is to study the baseline skin biophysical parameters and patch test results in individuals predisposing to XTT induced ICD.

**Methods:**

During a four-month period, 149 healthy volunteers with ICD and 50 volunteers without ICD after applying XTT were recruited. The skin biophysical parameters were measured, and contact allergy to 20 common allergens was patch tested, at two weeks after the ICD was recovered.

**Results:**

There were no significant differences in age and sex between ICD and control groups. It was found that skin median melanin value (176.50 vs 189.50,* P *< 0.05, Mann-Whitney U-test) and erythema value (319.90 ± 70.49 vs 347.93 ± 84.55,* P *< 0.05, Independent-Samples T test) were much lower in ICD than control group. Overall patch test results were not different, but the positivity rate of nickel sulfate (15.44% vs 4.00%,* P *< 0.05, Fisher's exact test) was significantly higher in ICD group.

**Conclusions:**

In conclusion, people with nickel allergy, lower values of skin melanin, and erythema are predisposing to develop ICD.

## 1. Introduction

Irritant contact dermatitis (ICD) is a nonimmunologic skin reaction. Xiaotong Tiegao (XTT) is an ancient topical Tibetan medicine plaster which is widely used in China for the treatment of various painful conditions such as torsion, bruising, rheumatoid arthritis, and scapulohumeral periarthritis to relieve pain. XTT contains Lamiophlomis rotata, Oxytropis falcate Bunge, Curcuma longa Linn, and Myricaria bracteata, which has shown a prominent anti-inflammatory effect [1]. To relieve pain, XTT is applied on the skin of pain area and stays for 24 hours; then it is removed and replaced with another one. Although XTT is very effective, the skin irritation caused by XTT is very common [2, 3]. Because the skin reaction caused by XTT can be reduced or completely avoided by shortening the applying time and, in patients with skin reactions, XTT can be reused without any new reaction several days later when the dermatitis disappeared (unpublished data), the dermatitis caused by XTT is ICD [4]. In clinics, it will be very useful to predict who may develop ICD before applying XTT. However, it is still unknown why some people are more predisposing to develop ICD under the same condition. Transepidermal water loss (TEWL) has been studied in 1020 male patients with irritant hand dermatitis, and it was found that the baseline TEWL could not be used as predictive indicator for the development of ICD [5]. Skin hydration, TEWL, sebum content, and pH have been compared between individuals with and without irritation responses after applying lactic acid, and it was found that TEWL was elevated in the irritated skin; however, the baseline TEWL between these two groups was not reported [6].

It is well known that nickel allergy is common in ICD individuals [7], while contact allergy in individuals predisposing to XTT induced ICD is still unknown. In this study, the baseline of skin biophysical parameters and contact allergy to 20 common contact allergens between the individuals with XTT induced ICD and those without were investigated.

## 2. Subjects and Methods

### 2.1. Induction of ICD by XTT in Healthy Individuals

Healthy adult volunteers were recruited from our hospital staff; volunteers aged above 80 years, experiencing any skin diseases, or taking immunosuppressant or steroids within the latest four weeks were excluded. Knowledge or suspicion of an existing allergy was neither an inclusion nor an exclusion criterion. XTT (Tibet Linzhi Cheezheng Tibetan Medicine Factory, Lanzhou, China) were applied on the back or limbs for 5 days; if the dermatitis was developed, the plaster was discontinued, and, after 2 weeks, when the dermatitis disappeared, XTT was reapplied to exclude allergic contact dermatitis.

The severity of skin reaction was evaluated according to the following criteria: (i) mild erythema and infiltration, possibly papules; (ii) moderate erythema and infiltration with papules and vesicles; (iii) severe erythema and infiltration with coalescing vesicles. The onset day of skin reaction was also recorded.

All participants were Han Chinese. The study was approved by the institutional review board of the Beijing Friendship Hospital Ethics Committee. All volunteers were provided written informed consent before participating in the study. The study was in accordance with principles of the Helsinki Declaration.

### 2.2. Detecting of Skin Biophysical Parameters

The CK Multi-Probe Adapter 580 (Courage+Khazaka Electronic GmbH, Germany) was used to measure the skin hydration, TEWL, elasticity, melanin value, erythema value, and pH. Biophysical parameters were measured on the forehead 1 cm above the center of bilateral eyebrows in a room with constant temperature and humidity after the volunteers quietly rested for 30 minutes. All of the measurements were performed by the same investigator. Measurement was performed three times consecutively and average was calculated.

### 2.3. Patch Testing

Patch testing was performed with 20 common allergens including 1% Cobalt chloride, 2% Mercapto mix, sulfur hydrogen mix, 2% Imidazolidinyl urea, 1% P-phenylenediamine, 1% N-cyclohexyl sulfur phthalide, 0.5% Potassium dichromate, 1% Ethylenediamine hydrochloride, 20% Rosin, 1% Formaldehyde, 1% Bisphenol-A epoxy resin, 0.25% Bronopol, 1% Thiuram mix, 16% Nipagin, 5% Nickel sulfate, 0.1% Sesquiterpenes, lactone mix, 5% Fragrance mix, 0.01% Methyl chloride isothiazolin, 0.6% Black rubber mix, 3% Carba mix, and 1% Quatermium-15 in IQ Chambers™ (Chemotechnique Diagnostics, Vellinge, Sweden). Allergens were applied to the upper back for two days, and the results were recorded on days D2 and D3, according to the guidelines of International Contact Dermatitis Research Group [8].

### 2.4. Statistical Analyses

The data were performed with SPSS17.0 (SPSS™ Statistics, Inc., Chicago, IL, USA). Mean ± standard deviation (SD) was expressed when the data distribution was Gaussian, and Independent-Samples T test was used to compare the differences; median was expressed when the data distribution was not Gaussian, and nonparametric Mann-Whitney U-test was used to compare the differences; Fisher's exact or the *χ*^2^ tests were used to compare the positive rate of patch test and the difference of age.* P *< 0.05 was considered statistically significant.

## 3. Results

From January 2016 to June 2016, 286 volunteers were screened for XTT induced dermatitis. The first 150 who developed dermatitis and 50 without any skin reaction were further studied. Of those 150 volunteers with dermatitis, only one developed dermatitis after reapplying XTT; therefore, 149 of them were considered having ICD. There were 118 females and 31 males, with an average age of 50.74 years (range 30–73 years). Of those 50 volunteers without any skin reaction, there were 34 females and 16 males, with an average age of 51.14 years (range 28–67 years).

**Figure 1:**
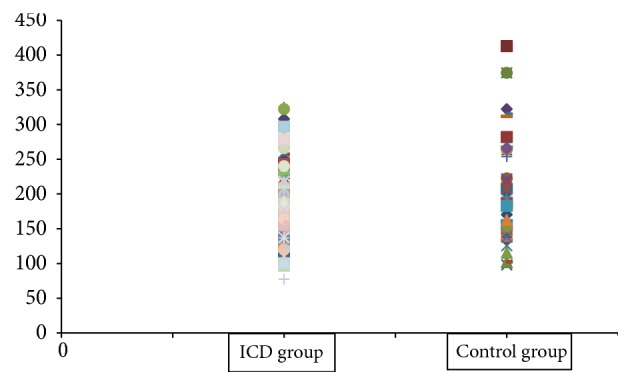
Melanin value between ICD and control groups.

**Figure 2:**
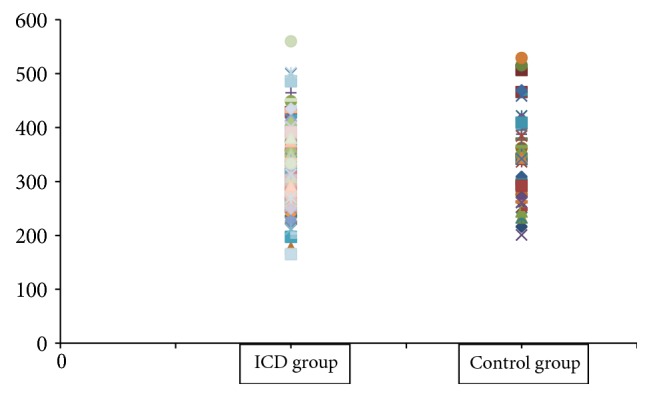
Erythema value between ICD and control groups.

Comparison of skin biophysical parameters between ICD group and control group is given in Table 1. The melanin value and erythema values were significantly higher in control group than ICD group (Table 1), and the scatter diagram was drawn to show the overlap interval of them in Figures 1 and 2. TEWL, skin elasticity, and skin pH were not different between two groups (Table 1).

70 out of 149 volunteers had mild ICD, 75 had moderate ICD, and only 4 had severe ICD. The comparison of skin biophysical parameters between mild ICD and moderate ICD is given in Table 2. Hydration in individuals with mild ICD was significantly higher than those with moderate ICD.

Skin biophysical parameters were also compared between individuals whose dermatitis appeared within two days and those more than two days; and no differences were found (Table 3).

The total positivity rates between individuals with ICD and those without were not different (*P *> 0.05). The positivity rate of nickel sulfate with ICD group was significantly higher than control group (Table 4).

The skin biophysical parameters were compared between individuals with positive response of nickel sulfate and those with negative response within ICD group, and skin hydration was significantly lower with individuals with negative response of nickel sulfate (Table 5).

## 4. Discussion

Based on the clinical manifestations and provocation testing results, the skin reaction caused by XTT is considered as ICD. However, ICD caused by XTT is different from other ICD. Although some individuals with ICD could reexpose to the irritants without any reaction later, this process is called “hardening phenomenon” [9], which usually takes a long time to achieve it. In our cases, the period of hardening was rather short, and most individuals could be tolerated to XTT in rechallenge test.

In our study, we found that the melanin and erythema values were lower in individuals who developed skin reaction after applying the XTT. It is well-known that people with darker skin are better protected from ultraviolet radiation, but whether melanin can protect for ICD has not been studied. However, it has been shown that white people are more likely to develop ICD compared to black ones, and it was considered that black people had better barrier function with increased intercellular cohesiveness and higher lipid content of stratum corneum [10]. A retrospective study in 2017 also found that, among the production workers who developed ICD, white people composed the majority of those [11].

It has not been reported about erythema value in ICD; since the erythema value is the symbol of blood flow, we consumed that better local blood circulation might protect the skin from irritants. However, there was great large overlap in values of melanin and erythema between the individuals with ICD and control group, and it is hard to use these parameters to predict ICD.

Our results further confirmed that though TEWL value is higher in ICD patients during irritation period, there was no difference in the baseline of TEWL between control group and ICD, nor was there a difference in the skin hydration, skin pH, and elasticity values.

There were no differences between two groups in overall positivity rate to 20 common allergens (Table 4). However, the positivity rate of nickel sulfate in ICD group was significantly higher than control group. Our results were consistent with previous study that nickel sulfate was the most common allergen among occupational ICD [12]. Among healthcare workers with ICD, nickel sulfate was also found out to be the most common allergen [7].

Increasing TEWL and decreasing skin hydration have been widely used for the measurement of skin irritation; however, the relationship between baseline skin hydration and irritation has not been reported. In this study, the value of skin hydration was higher in individuals with mild ICD and positive nickel allergy, but TEWL was not different; the relationship between them should be studied further.

There are some limitations about this study; whether the results are applicable to other irritants is unknown. We studied ICD induced by XTT; more researches need to be conducted to find optimum solution to ICD population.

## Figures and Tables

**Table 1:** Skin biophysical parameters between ICD and control groups.

	ICD group	Control group	*P*
	(N=149)	(N=50)	
Age (years)	50.74 ±7.03	51.14 ±7.75	0.73
Sex	Female:Male (118:31)	Female:Male (34: 16)	0.11
Skin hydration	52.86±14.25	48.93±13.86	0.09
TEWL (g/hm^2^)	20.67	22.92	0.14
Skin elasticity	0.62±0.09	0.62±0.09	0.85
Melanin value	176.50	189.50	*0.048*
Erythema value	319.90±70.49	347.93±84.55	*0.02*
Skin pH	5.73	5.76	0.89

Mean ± standard deviation (SD) was expressed when the data distribution was Gaussian; median was expressed when the data distribution was not Gaussian: TEWL (transepidermal water loss) and skin pH.

**Table 2:** Skin biophysical parameters between mild and moderate ICD.

	Mild ICD	Moderate ICD	*P*
	(N=70)	(N=75)	
Age (years)	49.93±6.79	51.63±7.28	0.15
Sex	Female:Male (57:13)	Female:Male (57:18)	0.43
Skin hydration	57.12±13.12	49.34±14.26	*0.001*
TEWL (g/hm^2^)	21.47	19.1	0.16
Skin elasticity	0.62±0.09	0.61±0.08	0.44
Melanin value	176.25	176.00	0.65
Erythema value	319.82±67.76	319.45±75.16	0.98
Skin pH	5.79	5.68	0.42

Mean ± standard deviation (SD) was expressed when the data distribution was Gaussian; median was expressed when the data distribution was not Gaussian: TEWL (transepidermal water loss) and skin pH.

**Table 3:** Skin biophysical parameters between individuals whose dermatitis appeared within two days and those more than two days.

	≤two days	>two days	*P*
	(N=81)	(N=68)	
Age (years)	50.32 ±6.94	51.24±7.15	0.43
Sex	Female:Male (67:14)	Female:Male (51:17)	0.25
Skin hydration	54.29±13.82	51.16±14.67	0.18
TEWL (g/hm^2^)	19.20	21.00	0.32
Skin elasticity	0.62±0.09	0.61±0.08	0.29
Melanin value	176.00	177.75	0.62
Erythema value	303.33	326.00	0.26
Skin pH	5.76	5.72	0.62

Mean ± standard deviation (SD) was expressed when the data distribution was Gaussian; median was expressed when the data distribution was not Gaussian: TEWL (transepidermal water loss) and skin pH.

**Table 4:** Patch test results n/(%) between ICD and control groups.

Allergens	ICD group	Control group	*P*
	n=149	n=50	
1% Cobalt chloride	1	2	0.16
2% Mercapto mix; sulfur hydrogen mix	0	1	0.25
2% Imidazolidinyl urea	0	0	
1% P-phenylenediamine	2	2	0.56
1% N-cyclohexyl sulfur phthalide	0	0	
0.5% Potassium dichromate	1	0	1
1% Ethylenediamine hydrochloride	1	1	0.44
20% Rosin	0	0	
1% Formaldehyde	1	0	1
1% Bisphenol-A epoxy resin	0	0	
0.25% Bronopol	0	0	
1% Thiuram mix	0	1	0.25
16% Nipagin	1	0	1
5% Nickel sulfate	23(15.44%)	2(4.00%)	*0.04*
0.1% Sesquiterpenes; lactone mix	1	0	1
5% Fragrance mix	8	3	1
0.01% Methyl chloride isothiazolin	1	0	1
0.6% Black rubber mix	1	1	0.44
3% Carba mix	0	0	
1% Quatermium-15	0	0	

**Table 5:** Skin biophysical parameters between individuals with positive response of nickel and those without of ICD group.

	Positive response of nickel (n=23)	Negative response of nickel (n=126)	*P*
Age (years)	50.00	52.00	0.70
Sex	Female:Male (22:1)	Female:Male (96:30)	0.07
Skin hydration	59.17±12.39	51.71±14.31	*0.02*
TEWL (g/hm^2^)	16.63	20.89	0.11
Skin elasticity	0.61±0.07	0.62±0.09	0.45
Melanin value	176.00	177.00	0.15
Erythema value	303.10±58.04	322.97±72.32	0.22
Skin pH	5.82	5.73	0.74

Mean ± standard deviation (SD) was expressed when the data distribution was Gaussian; median was expressed when the data distribution was not Gaussian: TEWL (transepidermal water loss) and skin pH.

## Data Availability

The data supporting the results of this study are included within the article, and original data are available from the corresponding author if needed.
